# A high-resolution daily gridded meteorological dataset for Serbia made by Random Forest Spatial Interpolation

**DOI:** 10.1038/s41597-021-00901-2

**Published:** 2021-04-30

**Authors:** Aleksandar Sekulić, Milan Kilibarda, Dragutin Protić, Branislav Bajat

**Affiliations:** University of Belgrade, Faculty of Civil Engineering, Department of Geodesy and Geoinformatics, Belgrade, 11000 Serbia

**Keywords:** Environmental sciences, Climate sciences

## Abstract

We produced the first daily gridded meteorological dataset at a 1-km spatial resolution across Serbia for 2000–2019, named MeteoSerbia1km. The dataset consists of five daily variables: maximum, minimum and mean temperature, mean sea-level pressure, and total precipitation. In addition to daily summaries, we produced monthly and annual summaries, and daily, monthly, and annual long-term means. Daily gridded data were interpolated using the Random Forest Spatial Interpolation methodology, based on using the nearest observations and distances to them as spatial covariates, together with environmental covariates to make a random forest model. The accuracy of the MeteoSerbia1km daily dataset was assessed using nested 5-fold leave-location-out cross-validation. All temperature variables and sea-level pressure showed high accuracy, although accuracy was lower for total precipitation, due to the discontinuity in its spatial distribution. MeteoSerbia1km was also compared with the E-OBS dataset with a coarser resolution: both datasets showed similar coarse-scale patterns for all daily meteorological variables, except for total precipitation. As a result of its high resolution, MeteoSerbia1km is suitable for further environmental analyses.

## Abstract (summary)

**Table:** 

Measurement(s)	temperature of air • pressure • volume of hydrological precipitation
Technology Type(s)	weather station
Factor Type(s)	digital elevation model (DEM) • topographic wetness index (TWI) • Tropical Rainfall Measuring Mission/Integrated Multi-satellitE Retrievals for Global Precipitation Measurement (TRMM/IMERG)
Sample Characteristic - Environment	climate
Sample Characteristic - Location	Serbia

Machine-accessible metadata file describing the reported data: 10.6084/m9.figshare.14102393

## Background & Summary

Daily meteorological observations are available from various sources, such as Global Historical Climate Network Daily (GHCN-daily)^1^, Global Surface Summary of the Day (GSOD)^2^, European Climate Assessment & Dataset (ECA&D)^3^, and OGIMET^4^. However, there is no information from these sources on daily meteorological variable values at unobserved locations, and so gridded meteorological datasets are made. Daily gridded meteorological datasets are essential input for numerous models and analyses across various research fields. For example, daily meteorological gridded datasets are used in agriculture for estimating yield^5,6^, the occurrence of insect pests and disease^7^, and crop growth^8^, as well as in meteorology^9^, hydrology^10^, ecology^11^, climate and climate change analysis^12^, risk assessment^13^, and forestry^14^.

Various sources of daily gridded meteorological datasets on global and regional levels cover the territory of Serbia. The details about these datasets are given in Table 1.

**Table 1:** Existing daily gridded meteorological datasets for Serbia (Ref. stands for reference and RS for remote sensing).

Dataset name	Abbreviation	Ref.	Dataset type	Spatial resolution
Moderate Resolution Imaging Spectroradiometer Land Surface Temperature	MODIS LST	^52^	RS-based	1 km
Tropical Rainfall Measuring Mission/Integrated Multi-satellitE Retrievals for Global Precipitation Measurement	TRMM/IMERG	^26^	RS-based	0.1° (~10 km)
Precipitation Estimation from Remotely Sensed Information using Artificial Neural Networks	PERSIANN	^53^	RS-based	0.04° (~4 km)
Climate Prediction Center global temperature and precipitation	CPC	^54,55^	station-based	0.5° (~50 km)
Ensembles daily gridded observational dataset	E-OBS	^27^	station-based	0.1° (~10 km)
Climate of the Carpathian region (covers only the northern part of Serbia)	CarpatClim	^56^	station-based	0.1° (~10 km)
National Centers for Environmental Prediction/National Center for Atmospheric Research reanalysis	NCEP/NCAR	^57^	reanalysis	2.5° (~250 km)
National Oceanic and Atmospheric Administration (NOAA) - CIRES 20th Century Reanalysis	NOAA-CIRES	^58^	reanalysis	2.5° (~250 km)
ERA-Interim	ERA-Interim	^59^	reanalysis	80 km
ERA5 (hourly, but can be aggregated to a daily resolution)	ERA5	^60^	reanalysis	0.25° (~25 km)

Most daily gridded datasets at the global and regional levels produced at a coarser spatial resolution can hardly represent localized meteorological patterns, which is their main limitation. MODIS LST has a finer spatial resolution (1 km), but daily products do not cover the entire spatial domain. Therefore, there is a need for localised meteorological gridded datasets at finer spatial resolutions. High-resolution daily gridded meteorological datasets are available for other regions^15–21^, but so far there has not been one for Serbia.

With this in mind, we developed the **MeteoSerbia1km** dataset, the first daily gridded (gap-free) meteorological dataset at a 1-km spatial resolution across Serbia, for the period 2000–2019. The MeteoSerbia1km dataset consists of daily maximum, minimum and mean temperatures (Tmax, Tmin, Tmean), the mean sea-level pressure (SLP), and the total precipitation (PRCP). The Random Forest Spatial Interpolation methodology (RFSI)^22^ was used for this purpose. RFSI was selected as it combines environmental covariates and observations from the nearest stations, in order to predict values at unobserved locations. Additionally, monthly and annual averages and daily, monthly, and annual long-term means (LTM) were made by averaging (or summing for PRCP) the MeteoSerbia1km dataset. The accuracy of the MeteoSerbia1km daily grids was assessed by nested k-fold cross-validation. Because there are no daily gridded meteorological datasets for Serbia and there is no reference point, MeteoSerbia1km was compared with the E-OBS daily gridded dataset at a spatial resolution of 10 km. MeteoSerbia1km was also tested with independent station observations.

As daily gridded meteorological datasets mostly cover a longer period of time, they can help in understanding the behaviour of meteorological variables in both spatial and temporal domains. The newly developed MeteoSerbia1km dataset is suitable for localized environmental and microclimate analyses, precision agriculture, forestry, regional and urban planning, hydrological analysis, and risk management in Serbia. The MeteoSerbia1km dataset is freely available in the GeoTIFF format. Daily products will be frequently updated. The dataset will also be improved in the future with further developments in the RFSI methodology, adding additional environmental covariates and including national meteorological observations.

## Methods

### Study area

**Fig. 1:**
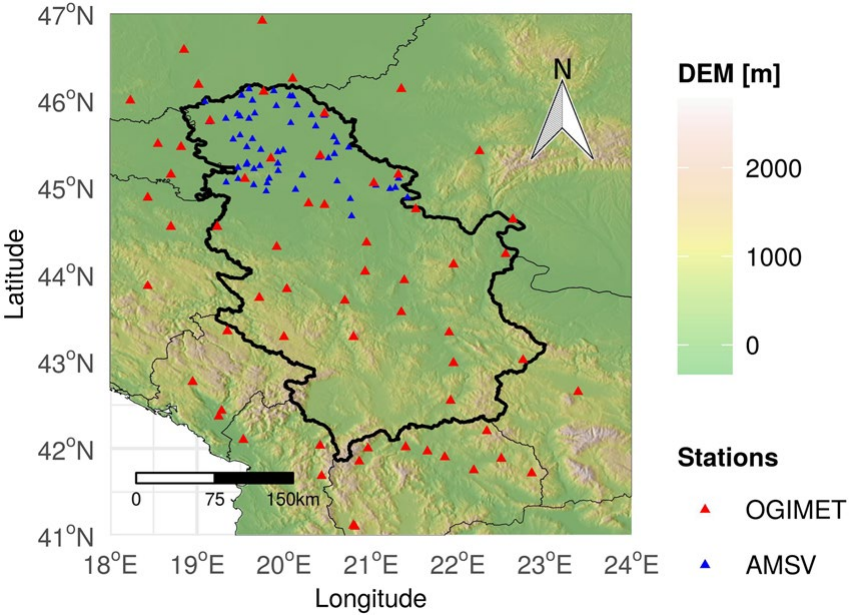
OGIMET and AMSV station locations used for making and testing MeteoSerbia1km with DEM.

Serbia is a medium sized Southeastern European country that covers an area of 88,361 km2, i.e., around 18% of the Balkan Peninsula (18.8°–23.0° E longitude, 41.8°–46.2° N latitude). It is characterized by a complex topography (Fig. 1, Digital Elevation Model (DEM)), since its northern parts are within the Pannonian Plain, and southern parts are crossed with several mountain systems. The mean altitude of Serbia is 473 m, ranging from 29 m in the northeast to 2,656 m on Prokletije Mountain in the southwest^23^. There are three main types of climate in Serbia, from north to south: continental, moderate continental, and modified Mediterranean climate. Precipitation is unevenly distributed with an average amount of 739 mm, and the average temperature for the period 1961–2010 was 10.4°C^24^.

### Observational source data

OGIMET and Automated meteorological stations in Vojvodina (AMSV) are two observational datasets from which daily meteorological variables from the OGIMET data were used as dependent variables in the modelling process, while AMSV data was used for evaluation of the MeteoSerbia1km dataset.

#### OGIMET

OGIMET^4^ is a Weather Information Service which provides data that includes historical daily summaries from surface synoptic observation (SYNOP) reports starting from the year 2000. SYNOP reports are meteorological alphanumeric messages for reporting observations from more than 10,000 meteorological stations around the world. Reports are mostly available every 6 h (00, 06, 12 and 18 UTC), but for some stations every 3 or 1 h. The format of these reports is standardized and defined by the World Meteorological Organization (WMO). OGIMET daily summaries from 61 SYNOP stations, of which 28 are in Serbia, were used for the spatial interpolation of meteorological variables (Fig. 1). The remaining 33 stations in a 100-km buffer around the Serbian border were used for a more accurate spatial interpolation, especially in the areas near the Serbian border.

The outliers for OGIMET precipitation daily summaries that were four times larger than (a) the maximum of the surrounding observations, i.e., observations in a radius of 100 km and (b) the corresponding E-OBS value (see section E-OBS) were detected and removed.

A summary of the statistics for each of the meteorological parameters is given in Table 2.

**Table 2:** Summary of the statistics for the selected variables in OGIMET daily summaries for the period 2000–2019.

Parameter	Tmax [°C]	Tmin [°C]	Tmean [°C]	SLP [mbar]	PRCP [mm]
Minimum	−22.2	−34.8	−24.8	967.4	0.0
1^st^ quartile	9.7	0.5	5.0	1,012.5	0.0
Median	18.3	6.9	12.3	1,016.5	0.0
Mean	17.6	6.4	11.8	1,017.1	2.0
3^rd^ quartile	25.8	12.7	18.9	1,021.4	1.0
Maximum	45.9	30.8	35.4	1,077.8	198.0

#### Automated meteorological stations in Vojvodina

AMSV^25^ collects hourly data for temperature (Tmax, Tmin, Tmean), the dew point, PRCP, relative humidity, etc., which began in March 2005. AMSV daily summaries from 55 stations (Fig. 1) were used to independently test MeteoSerbia1km in Vojvodina, specifically Tmax, Tmin, Tmean, and PRCP.

### Gridded source data

The DEM (Fig. 1), topographic wetness index (TWI) and IMERG gridded data were used as independent (auxiliary) variables (covariates) in the modelling process for the daily meteorological variables, while the E-OBS dataset was used for evaluation of the MeteoSerbia1km dataset.

#### DEM and TWI

A DEM at a spatial resolution of 1 km was created by combining SRTM 30 + and ETOPO DEM. A TWI at a spatial resolution of 1 km was derived from the SAGA GIS TWI algorithm and DEM. DEM and TWI both have a 1-km spatial resolution.

#### IMERG

IMERG^26^ is an algorithm that combines information from multiple sources, such as satellite microwave precipitation estimates, microwave-calibrated infrared satellite estimates, precipitation gauges, and other precipitation estimators to estimate precipitation over the majority of the Earth’s surface. One of the IMERG products is maps (grids) of daily precipitation estimates. The IMERG final run version V06B precipitation estimates were used for developing the PRCP model. IMERG estimates are a space-time covariate with a spatial resolution of 10 km and temporal resolution of one day. Earlier versions of the IMERG dataset, based on GPM, covered the period from 2014, but starting from version V06B, IMERG includes TRMM preprocessed data going back to June 2000. The IMERG dataset was used as a coarser scale covariate for precipitation. Therefore, the IMERG dataset was resampled to a 1-km spatial resolution using bilinear interpolation and DEM as a base layer.

#### E-OBS

E-OBS^27^ is an ensemble dataset constructed through a conditional simulation procedure. For each of the 100 members of the ensemble, a spatially correlated random field is produced using a pre-calculated spatial correlation function. The mean across the members is calculated and is provided as the “best-guess” fields. E-OBS is a daily dataset with a spatial resolution of 10 km. Because E-OBS is based on observations from ECA&D and SYNOP meteorological stations, it was used for comparison with the daily MeteoSerbia1km dataset and the detection of precipitation outliers.

### RFSI

RFSI^22^ is a novel methodology for spatial interpolation based on the random forest machine learning algorithm^28^. In comparison with other random forest models for spatial interpolation, RFSI uses additional spatial covariates: (1) observations at *n* nearest locations and (2) distances to them, in order to include the spatial context in the random forest. RFSI model predictions can be written as:

1 $$\widehat{z}({{\rm{s}}}_{0})=f({x}_{1}({{\rm{s}}}_{0}),\ldots ,{x}_{m}({{\rm{s}}}_{0}),z({{\rm{s}}}_{1}),{d}_{1},z({{\rm{s}}}_{2}),{d}_{2},z({{\rm{s}}}_{3}),{d}_{3},\ldots ,z({{\rm{s}}}_{n}),{d}_{n})$$

where $\widehat{z}({{\rm{s}}}_{0})$ is the prediction at prediction location s_0_, ${x}_{i}({{\rm{s}}}_{0})(i=1,\ldots ,m)$ are environmental covariates at location s_0_, *z*(s_*i*_) and *d*_*i*_ are spatial covariates (*i* = 1, …, *n*), where $z({{\rm{s}}}_{i})$ is the *i*-th nearest observation from s_0_ at location s_*i*_ and *d*_*i*_ = |s_*i*_ - s_0_|. These spatial covariates proved to be valuable extensions for the random forest algorithm in improving its spatial accuracy. A detailed description of RFSI, including its performance and implementation procedure, is provided by Sekulić *et al*.^22^.

#### Model development and prediction

In order to prepare the data for RFSI modelling, all of the environmental covariates were overlaid with training observation locations for each day. Then, RFSI spatial covariates were created in the following way: for each day and for each training observation location, *n* nearest training observation locations were found and *n* pairs of covariates—observations at *n* nearest locations and distances to them—were calculated. Extracted overlaid values and *n* pairs of spatial covariates were assigned to the corresponding observations, making a dataset which was then used to fit an RFSI model.

**Fig. 2:**
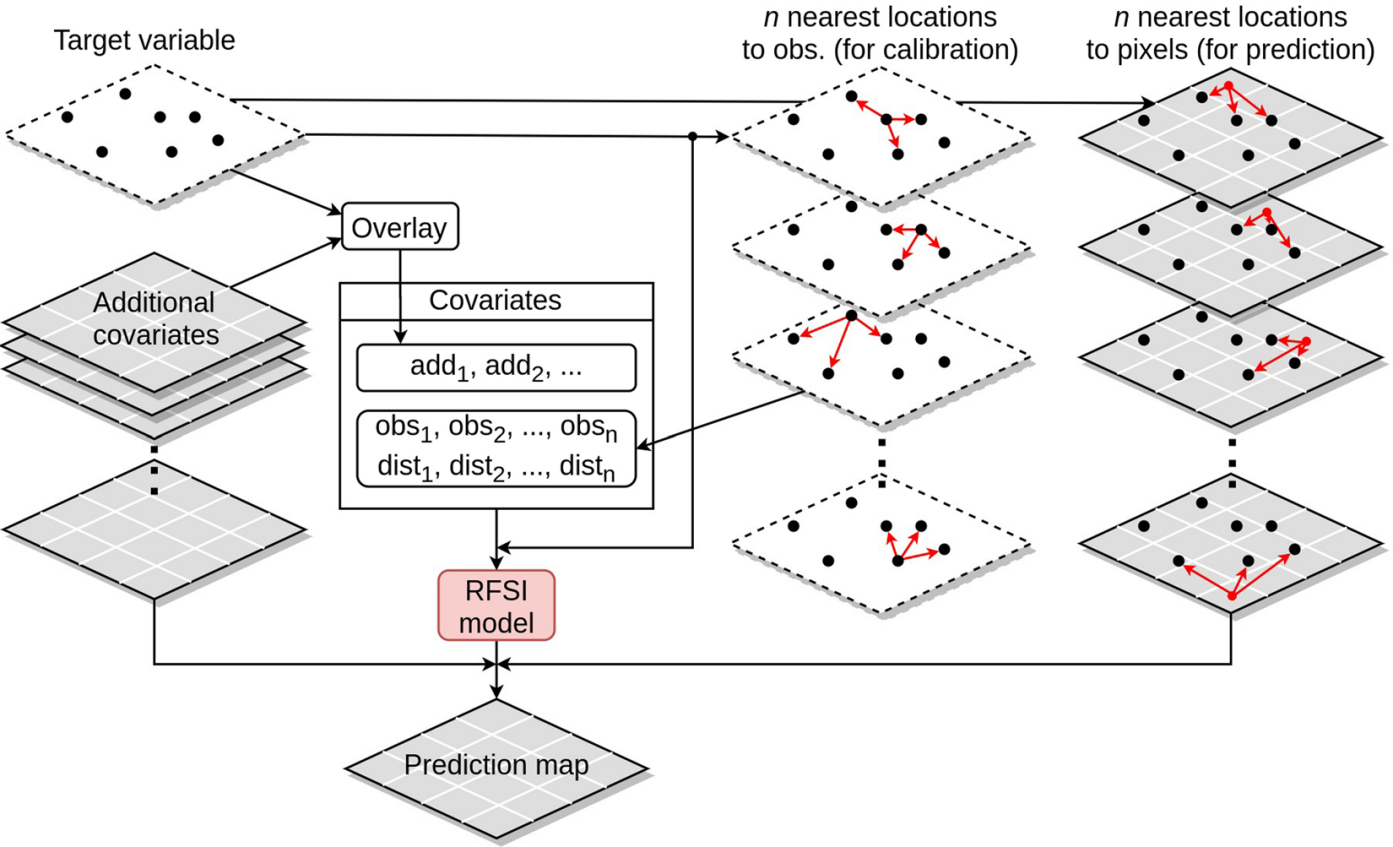
Schematic representation of the RFSI algorithm^22^.

Predictions were made in a similar manner to the development of the RFSI model. For each of the desired prediction days and locations (in this case pixels of the target grid), environmental covariates were extracted and observations at *n* nearest training locations and distances to them were calculated. Then, predictions were made using extracted values and *n* pairs of spatial covariates, and an already fitted RFSI model. The entire process of making the RFSI model and making predictions is presented in Fig. 2. It should be noted that the RFSI model can handle both regression and classification tasks.

#### Model tuning

In order to achieve the best possible prediction accuracy, hyperparameters for the RFSI models were tuned. The tuned hyperparameters were the number of variables to possibly split at each node (*mtry*), minimal node size (*min.node.size*) and ratio of observations-to-sample in each decision tree (*sample.fraction*), and the number of nearest observations (*n.obs*). The number of trees (*ntree*) hyperparameter was fixed and set to 250, according to Sekulić *et al*.^22^, as a larger value of *ntree* would not improve the RFSI model’s accuracy. The splitrule (*splitrule*) hyperparameter was also fixed and set as *variance* for regression tasks, and *gini* index for classification tasks.

The hyperparameters were tuned using 5-fold leave-location-out cross-validation (LLOCV). Here ‘leave-location-out’ means that all observations from a specific location (i.e. time series of observations from a station) were in the same fold, and 5-fold means that all of the locations were grouped into 5 groups (folds). Then, each of the folds was used once for validation. By doing so, the accuracy of the targeted spatial prediction was assessed^29^. Many different combinations of hyperparameters were tested and for each combination, 5-fold LLOCV was performed. In other words, for each of the hyperparameter combinations, the entire dataset was split into 5 folds. Each of the folds once represented a test fold, while the four remaining folds were used to fit the RFSI model with a hyperparameter combination. Finally, RMSE was adopted as a criterion for the selection of optimal hyperparameters. The RMSE was calculated for the entire dataset after the 5-fold LLOCV process, i.e., based on all observations and predictions from all 5 folds.

### Modelling of daily meteorological variables

#### Temperature

Modelling the daily temperature variables, Tmax, Tmin, and Tmean, is a regression task. All daily temperature RFSI models are as follows:

2 $${T}_{max,min,mean}({{\rm{s}}}_{0})={f}_{R}(DEM,TWI,GTT,DOY,IDW,z({{\rm{s}}}_{1}),{d}_{1},\ldots ,z({{\rm{s}}}_{n.obs}),{d}_{n.obs})$$

where *T*_*max,min,mean*_(s_0_) is the daily temperature (Tmax, Tmin, and Tmean) prediction at prediction location s_0_, *f*_*R*_ denotes an RFSI regression model, *GTT* is the geometrical temperature trend, a function of latitude and day of the year (which was shown to be a valuable covariate for Tmax, Tmin and Tmean)^30^, *DOY* is a temporal covariate, i.e., the day of the year, and *IDW* is a local inverse distance weighting prediction based on the *n.obs* number of nearest observations (excluding the observed location).

The tuned hyperparameters for each of the daily temperature models are given in Table 3. The *IDW* exponent (*p*) was also tuned. The *n.obs* hyperparameter was 10 for the Tmax model and 9 for the Tmin and Tmean models.

**Table 3:** Optimized hyperparameters for each of the daily meteorological variables.

Variable	mtry	min.node.size	sample.fraction	n.obs	p
Tmax	7	15	0.98	10	2.9
Tmin	4	11	0.93	9	2.2
Tmean	7	14	1.00	9	3.0
SLP	6	11	0.91	9	3.5
PRCP classification	3	2	0.70	9	n/a
PRCP regression	7	11	0.93	6	3.3

#### Sea-level pressure

Modelling the daily SLP is also a regression task. The SLP RFSI model has fewer covariates than corresponding temperature models:

3 $$SLP({{\rm{s}}}_{0})={f}_{R}(DEM,DOY,IDW,z({{\rm{s}}}_{1}),{d}_{1},\ldots ,z({{\rm{s}}}_{9}),{d}_{9})$$

where *SLP*(s_0_) is the daily SLP prediction at prediction location *s*_0_.

The tuned hyperparameters for the daily SLP model are given in Table 3. The *n.obs* hyperparameter was 9.

#### Precipitation

PRCP was modelled in two steps, i.e., with two models: (1) a classification model for the daily precipitation occurrence and (2) a regression model for the daily amount of precipitation, denoted as:

4 $$\begin{array}{l}PRCP({{\rm{s}}}_{0})={f}_{C}(DEM,{T}_{max},{T}_{min},{\rm{S}}LP,IMERG,DOY,z({{\rm{s}}}_{1}),{d}_{1},\ldots ,z({{\rm{s}}}_{9}),{d}_{9})\cdot \\ \quad \quad \quad {f}_{R}(DEM,{T}_{max},{T}_{min},{\rm{S}}LP,IMERG,DOY,IDW,z({{\rm{s}}}_{1}),{d}_{1},\ldots ,z({{\rm{s}}}_{6}),{d}_{6})\end{array}$$

where *PRCP*(s_0_) is the daily PRCP prediction at prediction location s_0_, *f*_*C*_ denotes the PRCP RFSI classification model with 0 and 1 as possible classes, *Tmax*, *Tmin*, and *SLP* are corresponding daily predictions from the MeteoSerbia1km dataset at location s_0_, and *IMERG* is the corresponding overlayed value from the IMERG dataset at location s_0_. Both precipitation models were fitted on the entire dataset with the same covariates. This means that zero precipitation observations were included in the regression model fitting. One reason for this was to include zero precipitation proximity in the regression model. As seen from Eq. 4, in PRCP prediction, the regression model was applied only in the locations where the classification model predicted the precipitation occurrence (class 1).

The tuned hyperparameters for both the daily PRCP classification and regression models are given in Table 3. The *n.obs* hyperparameter for the classification model was 9, and for the regression model was 6.

## Data Records

**Fig. 3:**
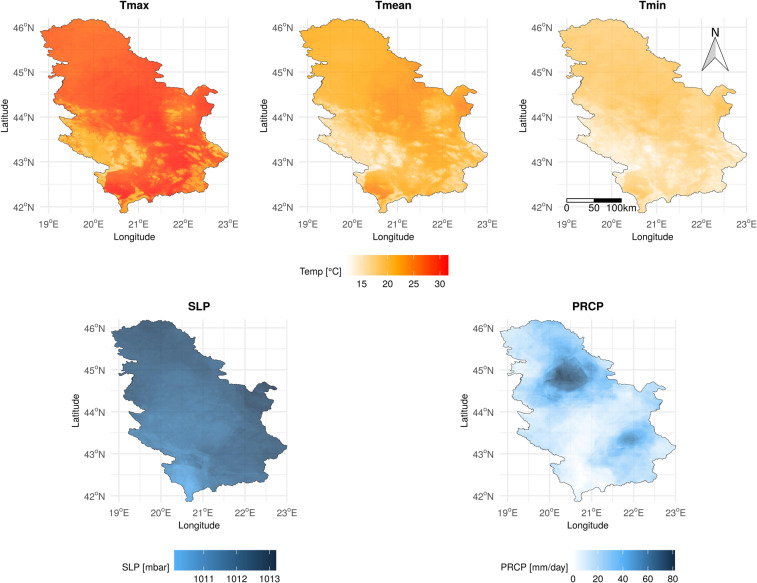
Prediction maps for all daily meteorological variables, for July 27, 2014.

MeteoSerbia1km is a high-resolution daily meteorological gridded dataset for Serbia, consisting of Tmean, Tmax, Tmin, SLP and PRCP variables, for the period 2000–2019. As an example, prediction maps for July 27, 2014 are presented in Fig. 3. In addition, monthly and annual averages (totals for PRCP) were generated by aggregating daily datasets. Then, daily, monthly, and annual LTM were generated by averaging daily, monthly and annual datasets. Since the first five months of the year 2000 were missing from the IMERG dataset, the daily and monthly PRCP averages start from June, 2000. Therefore, the daily and monthly PRCP LTMs were calculated without the first five months of the year 2000, and PRCP annual averages and LTM were calculated without the year 2000. Additionally, only the data for leap years were available for generating the daily LTM for February 29.

The OpenStreetMaps country border (https://osm-boundaries.com/https://osm-boundaries.com/) of Serbia was used to ensure that the MeteoSerbia1km dataset covers the territory of Serbia. The entire dataset is at a 1-km spatial resolution, and is available in both, WGS84 and UTM34N projections. The dataset is stored in the GeoTIFF (.*tif*) format. Units of the dataset values are

- temperature (Tmean, Tmax, and Tmin) - tenths of a degree in the Celsius scale (°C)
- SLP - tenths of a mbar
- PRCP - tenths of a mm

Furthermore, all dataset values are stored as integers (*INT*32 data type) in order to reduce the size of the GeoTIFF files, i.e., temperature values should be divided by 10 to obtain degrees Celsius, and the same for SLP and PRCP to obtain millibars and millimeters.

The file naming convention adopted is provided in Table 4. It should be noted that the naming convention is different for different products with different temporal resolutions.

**Table 4:** MeteoSerbia1km dataset file naming convention.

Product	File nomenclature	Example
Daily averages	var_{time period}_{yyyymmdd}_{proj}.tif	tmax_day_20000101_wgs84.tif
Monthly averages	var_{time period}_{yyyymm}_{proj}.tif	tmax_mon_200001_wgs84.tif
Annual averages	var_{time period}_{yyyy}_{proj}.tif	tmax_ann_2000_wgs84.tif
Daily LTM	var_ltm_{time period}_{mmdd}_{proj}.tif	tmax_ltm_day_0101_wgs84.tif
Monthly LTM	var_ltm_{time period}_{mm}_{proj}.tif	tmax_ltm_mon_01_wgs84.tif
Annual LTM	var_ltm_{time period}_{proj}.tif	tmax_ltm_ann_wgs84.tif

The dataset can be downloaded from ZENODO^31^ (10.5281/zenodo.4058167), year by year.

## Technical Validation

### Validation of daily datasets

The daily MeteoSerbia1km dataset was validated using nested 5-fold LLOCV, which combines nested k-fold^32^ and leave-location-out cross-validation. For nested 5-fold LLOCV, as with the regular 5-fold LLOCV, the entire dataset was split into five folds. Each of the folds was used once for testing, while the four remaining folds were used for hyperparameter tuning with regular 5-fold LLOCV (see the Model tuning section). Four accuracy metrics, namely, the coefficient of determination (R^2^), Lin’s concordance correlation coefficient (CCC)^33^, the mean absolute error (MAE) and the root mean square error (RMSE) were calculated for all daily meteorological variables for the stations in Serbia (Table 5). Note that the coefficient of determination represents the amount of variance explained by the model:

5 $${R}^{2}=1-\frac{ESS}{TSS}=1-\frac{{\sum }_{i=1}^{n}{(z({s}_{i})-\widehat{z}({s}_{i}))}^{2}}{{\sum }_{i=1}^{n}{(z({s}_{i})-\bar{z}({s}_{i}))}^{2}}$$

where *ESS* is the Error Sum of Squares, *TSS* the Total Sum of Squares, and $\bar{z}({s}_{i})$ the mean of the observations. The SLP model had the highest accuracy, especially for stations in Serbia, followed by Tmax and Tmean. This is due to the fact that SLP and temperature are continuous variables and have strong spatial autocorrelation. Tmin showed slightly lower accuracy than Tmax and Tmean, and PRCP showed the lowest accuracy, which has also been reported in similar studies^27,34^. Furthermore, LLOCV accuracy is lower for stations outside of Serbia because of the well-known edge effect interpolation problem. Therefore, including stations outside of Serbia in LLOCV would not give an objective accuracy assessment of the MeteoSerbia1km dataset and would even reduce the accuracy.

**Table 5:** Accuracy metrics for each meteorological variable for stations in Serbia, as assessed using the nested 5-fold LLOCV.

Variable	R^2^ [%]	CCC	MAE	RMSE
Tmax	97.4	0.987	1.1 °C	1.7°C
Tmin	93.7	0.968	1.4 °C	2.0°C
Tmean	97.4	0.987	1.0 °C	1.4°C
SLP	99.1	0.996	0.5 mbar	0.7 mbar
PRCP	63.8	0.784	1.1 mm	3.1 mm

The accuracy of both the two-step PRCP model with classification and the unique PRCP regression model was the same. The advantage of the PRCP two-step model with classification is that zero PRCP values were predicted as exact zeros. Cohen’s kappa coefficient^35^ for the PRCP RFSI classification in Serbia was 0.779. The confusion matrix is shown in Table 6. In cases where the observed values were zero (class 0), only 4.21% of the final predicted values were larger than 1 mm, and 0.44% of them were larger than 5 mm. If the opposite case was true, in which the predicted values were zero (class 0), only 3.94% of the observed values were larger than 1 mm, and 0.86% of them were larger than 5 mm.

**Table 6:** Confusion Matrix for the PRCP RFSI classification model from the nested 5-fold LLOCV.

		Observation	
		0	1
**Prediction**	0	108,248 (93.40%)	11,591 (16.35%)
	1	7,651 (6.60%)	59,298 (83.65%)

Class 0 represents no precipitation, and class 1 represents precipitation occurrence.

**Fig. 4:**
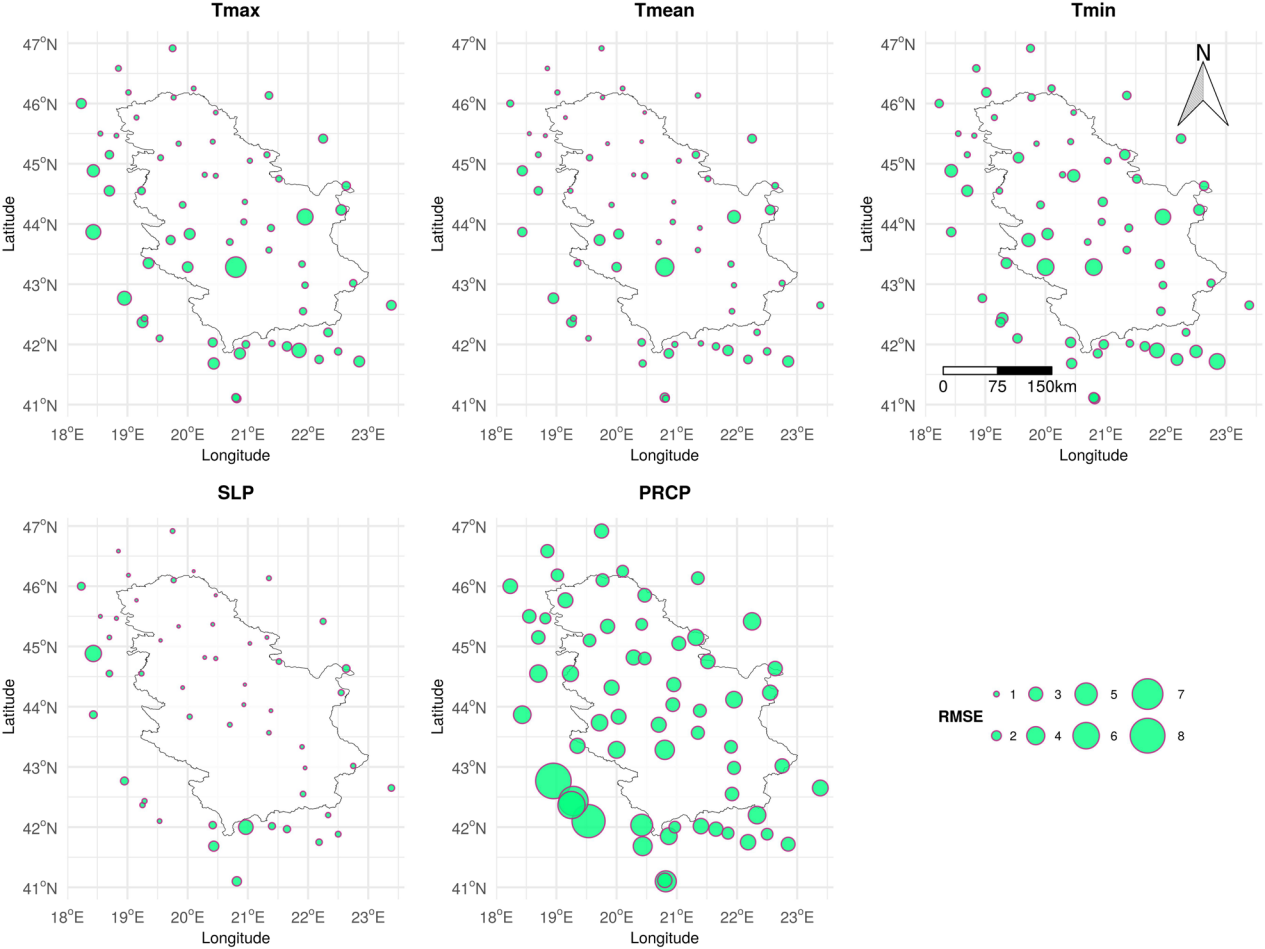
Average RMSE per station for the period 2000–2019, calculated from the nested 5-fold LLOCV. The units are °C for temperature, mbar for SLP and mm for PRCP.

**Fig. 5:**
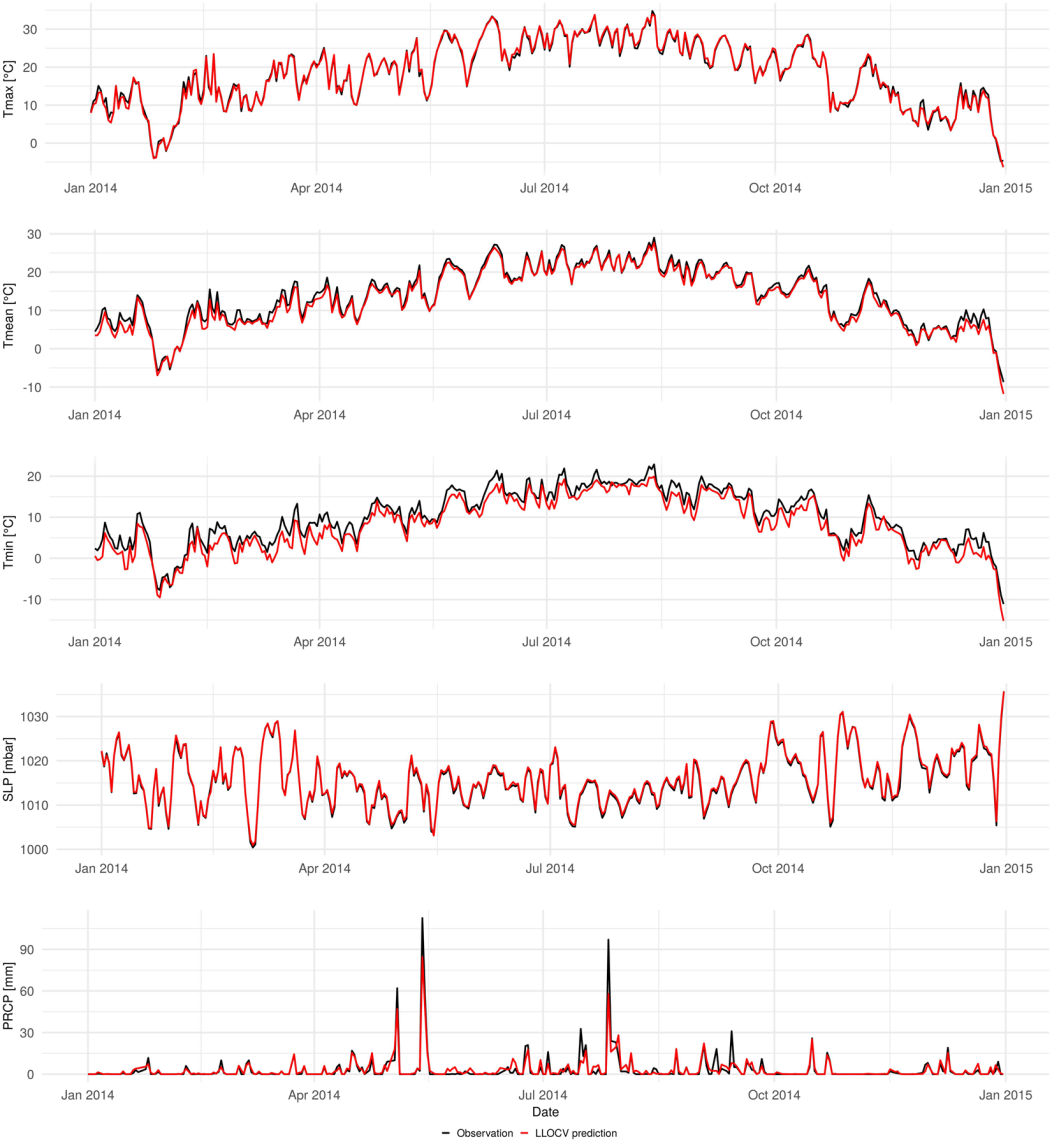
Predictions from the nested 5-fold LLOCV (red) and observations (black) for the Belgrade station for 2014.

The average RMSE per station for the entire time period is presented in Fig. 4. Stations at the highest altitudes, Kopaonik (1,711 m) and Crni Vrh (1,037 m), had the largest average RMSE for all temperature variables. Additionally, Sjenica (1,038 m) and Zlatibor (1,029 m) had a high average RMSE for Tmin, which is the reason for the lower accuracy in comparison with Tmax and Tmean. On the one hand, microclimatic conditions at higher altitudes affect the temperature behaviour, so that overall spatial autocorrelation, and therefore the accuracy, is lower. On the other hand, the accuracy is higher at lower altitudes, especially in Vojvodina, the northern part of Serbia. This makes temperature datasets particularly suitable for agriculture. The average RMSE for SLP is low and equally distributed for the territory of Serbia, which is confirmed by the overall high accuracy (Table 5). The average RMSE for PRCP is also equally distributed over the territory of Serbia. The time series of predictions from the nested 5-fold LLOCV and observations for the Belgrade station, for 2014, are presented in Fig. 5. The figure shows that differences between observations and predictions for Tmax, Tmean, and SLP are minor, whereas those for Tmin are somewhat larger, mostly because Tmin is slightly underestimated, as reflected in the lower accuracy in comparison with Tmax and Tmean (Table 5). For PRCP, the days without precipitation are predicted well, whereas the days with precipitation are slightly underestimated.

### Comparison with E-OBS

**Fig. 6:**
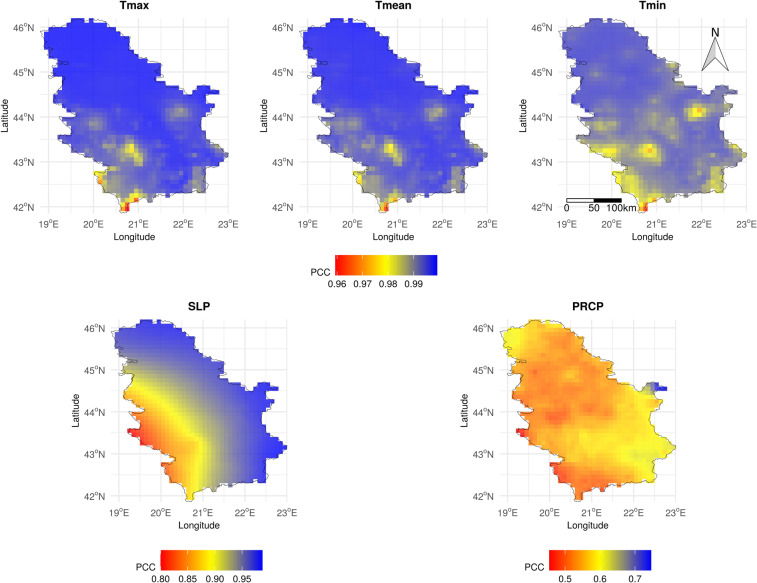
Pearson correlation coefficient map between E-OBS and the daily MeteoSerbia1km datasets for Serbia.

The E-OBS dataset was taken as a benchmark dataset because it was made by geostatistical simulation, i.e., spatial interpolation from ECA&D stations, which also includes SYNOP stations. The daily MeteoSerbia1km dataset was aggregated to a 10-km spatial resolution in order to match the pixels (grid) of the E-OBS gridded dataset. Then, for each of the raster pixels, a Pearson correlation coefficient (PCC) was calculated between the pixel time series of MeteoSerbia1km estimations and the pixel time series of E-OBS estimations. Maps of the PCCs for each meteorological variable are presented in Fig. 6.

The MeteoSerbia1km dataset shows an overall high correlation with the E-OBS dataset for Tmax, Tmin, Tmean, and SLP (0.992, 0.989, 0.993, and 0.922 respectively) and similar coarse-scale spatial patterns, with slightly lower correlation around the Kopaonik and Crni Vrh stations (Fig. 6), where the LLOCV accuracy was the lowest (Fig. 4). The correlation for SLP was lower in the southwestern part of Serbia, probably because of the lack of SYNOP SLP stations in that area (Fig. 4). The MeteoSerbia1km dataset showed the lowest correlation with the E-OBS dataset for PRCP (0.551). The main reason for this is that precipitation is a complex variable, and different models can produce significantly different results. Another reason is that the E-OBS methodology does not include IMERG, which is an important predictor for the PRCP model and, consequently, predictions follow IMERG patterns. Bearing in mind that the accuracy of MeteoSerbia1km and E-OBS PRCP models does not differ much in RMSE and MAE, RFSI PRCP can be valuable for the areas where E-OBS cannot contribute or where a finer spatial resolution of 1 km is needed. Hence, the MeteoSerbia1km dataset describes the local variation of daily PRCP in Serbia better than E-OBS.

### Test with stations in Vojvodina

MeteoSerbia1km was also tested with independent AMSV stations that were not used for making RFSI models. The RMSE between AMSV stations and the corresponding MeteoSerbia1km values over Vojvodina for the period 2005–present period for Tmax, Tmin, Tmean, and PRCP was 1.6°C, 1.8°C 1.2°C, and 3.7 mm, respectively. In comparison with the results from LLOCV for the whole of Serbia (Table 5), the accuracy of MeteoSerbia1km temperature variables is slightly better, while the accuracy of MeteoSerbia1km PRCP is slightly worse. Lower RMSE for PRCP can be taken as a consequence of a denser network of AMSV stations than OGIMET stations and a large spatial variability of PRCP.

## Usage Notes

MeteoSerbia1km is the first high-resolution daily gridded meteorological dataset for Serbia at a 1-km spatial resolution. The dataset can be used in a wide range of areas such as agriculture, insurance, forestry, climatology, meteorology, hydrology, ecology, soil mapping, urban planning, or any other research field that needs gridded data with a high spatial resolution.

MeteoSerbia1km is in the GeoTIFF format which makes it interoperable with any GIS software, such as SAGA GIS (http://www.saga-gis.org/), QGIS (http://www.qgis.org), ArcGIS (https://www.arcgis.com/), etc. It should be noted that MeteoSerbia1km values are multiplied by 10, so they should be divided by 10 to obtain values in basic units (°C, mbar and mm). Finally, the predictions for some days may show artifacts due to misrepresentation by meteorological stations.

The data are freely available under Creative Commons Licence: CC BY 4.0.

## Data Availability

The *R* programming language^36^, version 3.6.1, was used for the automation of the entire process for making the MeteoSerbia1km dataset, using the following packages: *climate*^37^, *meteo*^30^, *nabor*^38^, *CAST*^39^, *caret*^40^, *sp*^41,42^, *spacetime*^42,43^, *gstat*^44,45^, *raster*^46^, *rgdal*^47^, *doParallel*^48^, *ranger*^49^, *plyr*^50^, *ggplot2*^51^. To automate the development, tuning, cross-validation and prediction processes for the RFSI method, five additional *R* functions were created and added to the *R meteo* package^30^ (https://github.com/AleksandarSekulic/Rmeteo, http://r-forge.r-project.org/projects/meteo): • near.obs - for finding *n* nearest observations and distances to them from desired locations, • rfsi - for RFSI model fitting, • tune.rfsi - for RFSI model tuning, • cv.rfsi - for RFSI model cross-validation, • pred.rfsi - for RFSI model prediction. In order to make this work reproducible, a complete script in *R* and datasets used for the modelling, tuning, validation, and prediction of daily meteorological variables is available via the GitHub repository at https://github.com/AleksandarSekulic/MeteoSerbia1km.
